# Energy Stores Are Not Altered by Long-Term Partial Sleep Deprivation in *Drosophila melanogaster*

**DOI:** 10.1371/journal.pone.0006211

**Published:** 2009-07-10

**Authors:** Susan T. Harbison, Amita Sehgal

**Affiliations:** Howard Hughes Medical Institute, Department of Neuroscience, School of Medicine, University of Pennsylvania, Philadelphia, Pennsylvania, United States of America; Center for Genomic Regulation, Spain

## Abstract

Recent human studies reveal a widespread association between short sleep and obesity. Two hypotheses, which are not mutually exclusive, might explain this association. First, genetic factors that reduce endogenous sleep times might also impact energy stores, an assertion that we confirmed in a previous study. Second, metabolism may be altered by chronic partial sleep deprivation. Here we address the second assertion by measuring the impact of long-term partial sleep deprivation on energy stores using *Drosophila* as a model. We subjected flies to long-term partial sleep deprivation via two different methods: a mechanical stimulus and a light stimulus. We then measured whole-body triglycerides and glycogen, two important sources of energy for the fly, and compared them to un-stimulated controls. We also measured changes in energy stores in response to a random circadian clock shift. Sex and line-dependent alterations in glycogen and/or triglyceride levels occurred in response to the circadian clock shift and in flies subjected to a single night of sleep deprivation using light. Thus, consistent with previous studies, our findings suggest that acute sleep loss and changes to the circadian clock can alter metabolism. Significant changes in energy stores were also observed when flies were subjected to chronic sleep loss via the mechanical stimulus, although not the light stimulus. Interestingly, mechanical stimulation resulted in the same change in energy stores even when it was not associated with sleep deprivation, suggesting that the changes are caused by stress rather than sleep loss. These findings emphasize the importance of taking stress into account when evaluating the relationship between sleep loss and metabolism.

## Introduction

Recent human studies have discovered a widespread association between short sleep times, obesity, and diabetes (reviewed in [1]). These studies compare the body-mass index (BMI), which is body weight in kilograms divided by the square of height in meters, to the amount of time spent sleeping. A significant association between short sleep times (less than six hours per night) and high BMI is consistently reported in both male and female adults [2]–[8]. In some cases BMI forms a U-shaped distribution with sleep times; high BMI is associated with both short and long (nine hours or more per night) sleep times [4], [5], [8], [9]. In general, effect sizes tend to be small, but the association of short sleep with obesity has been consistently reported in human studies totaling over one million participants. Short sleep was also positively correlated with body fat [10] and non-fasted serum triglycerides, although the association was dependent upon gender, smoking, and BMI [8]. Thus, a growing body of evidence links metabolic disorders to sleep behavior.

However, endogenous sleep in humans is confounded with the voluntary curtailment of sleep [11]. Recent surveys by the National Sleep Foundation indicate that many Americans curb nightly sleep by two hours or more in favor of other activities [12], [13]. When humans are deprived of sleep experimentally, decreases in the appetite-suppressing hormone leptin [14]–[16] and increases in the appetite-stimulating hormone ghrelin [16] are observed, which may alter eating behavior in favor of weight gain. However, reduced leptin and increased ghrelin have also been linked with short sleep in the absence of experimental sleep deprivation [9], underscoring the difficulty in dissociating sleep curtailment from naturally short sleep times in humans.

It may be possible to use animal model systems to distinguish the metabolic effects of endogenous sleep need from those of chronic sleep deprivation. Indeed, we recently used *Drosophila melanogaster*, a model for mammalian sleep[17]–[19], to identify genetic correlations between normal sleep periods and metabolism [20]. Here we consider the metabolic impact of chronic partial sleep deprivation. We deprived flies of two hours of sleep every night for a week to mimic human voluntary sleep restriction. We used two methods, a mechanical stimulus and a light stimulus, to deprive flies of sleep. The light stimulus allowed us to distinguish between effects that were due to sleep loss and effects that were due to physical stimulation. To decouple the effect of the light stimulus on sleep from its effect on the molecular circadian clock, we examined the metabolic effects of a random circadian clock shift. Finally, to account for possible adaptation to the light stimulus, we measured the metabolic effects a single night of sleep loss induced with light.

We used triglyceride and glycogen levels to assess metabolic changes in sleep-deprived flies. Many genes involved in the storage of triglycerides in flies have been evolutionarily conserved in mammals, making triglyceride stores a relevant model for human systems[21]. Furthermore, both triglyceride and glycogen stores are important for stress resistance in flies [22]. Since our hypothesis was that sleep restriction would impact energy stores, flies were measured immediately after the sleep deprivation protocol on the last day to prevent recovery sleep. We compared triglyceride and glycogen levels of sleep-deprived flies to that of controls.

Our findings suggest that a single night of sleep loss and changes in the circadian clock can alter metabolism, consistent with human studies; however, chronic sleep loss in the absence of a physical stressor does not impact energy stores. On the other hand, physical stress impacts energy stores, suggesting that it should be considered as a contributing factor in all studies of short sleep times.

## Results

### Protocol for long-term partial sleep loss

**Figure 1:**
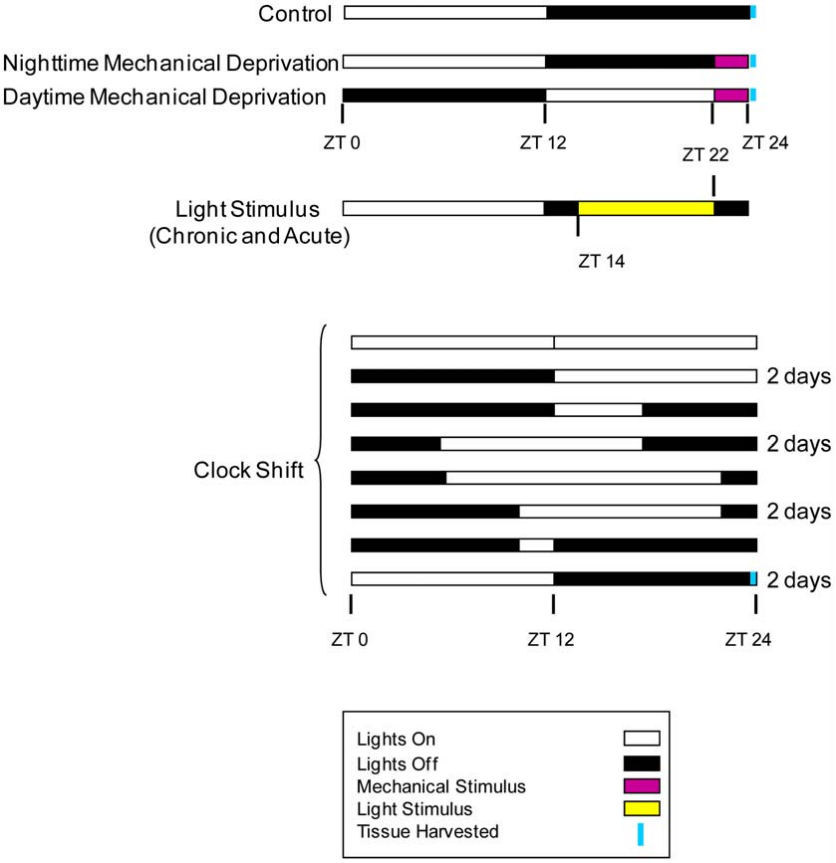
Sleep deprivation protocol used for the sleep deprivation experiments. The bars indicate the light∶dark cycles experienced by the flies, with white signifying the light period, and black signifying the dark period. Light∶dark cycles are aligned to the control light∶dark cycle. The mechanical stimulus and chronic light stimulus experiments were conducted over a seven-day period. The acute light stimulus experiment lasted 24 hours. The constant clock shift experiment lasted 12 days; some light∶dark patterns lasted two days, as indicated. ZT, zeitgeber time.

**Figure 2:**
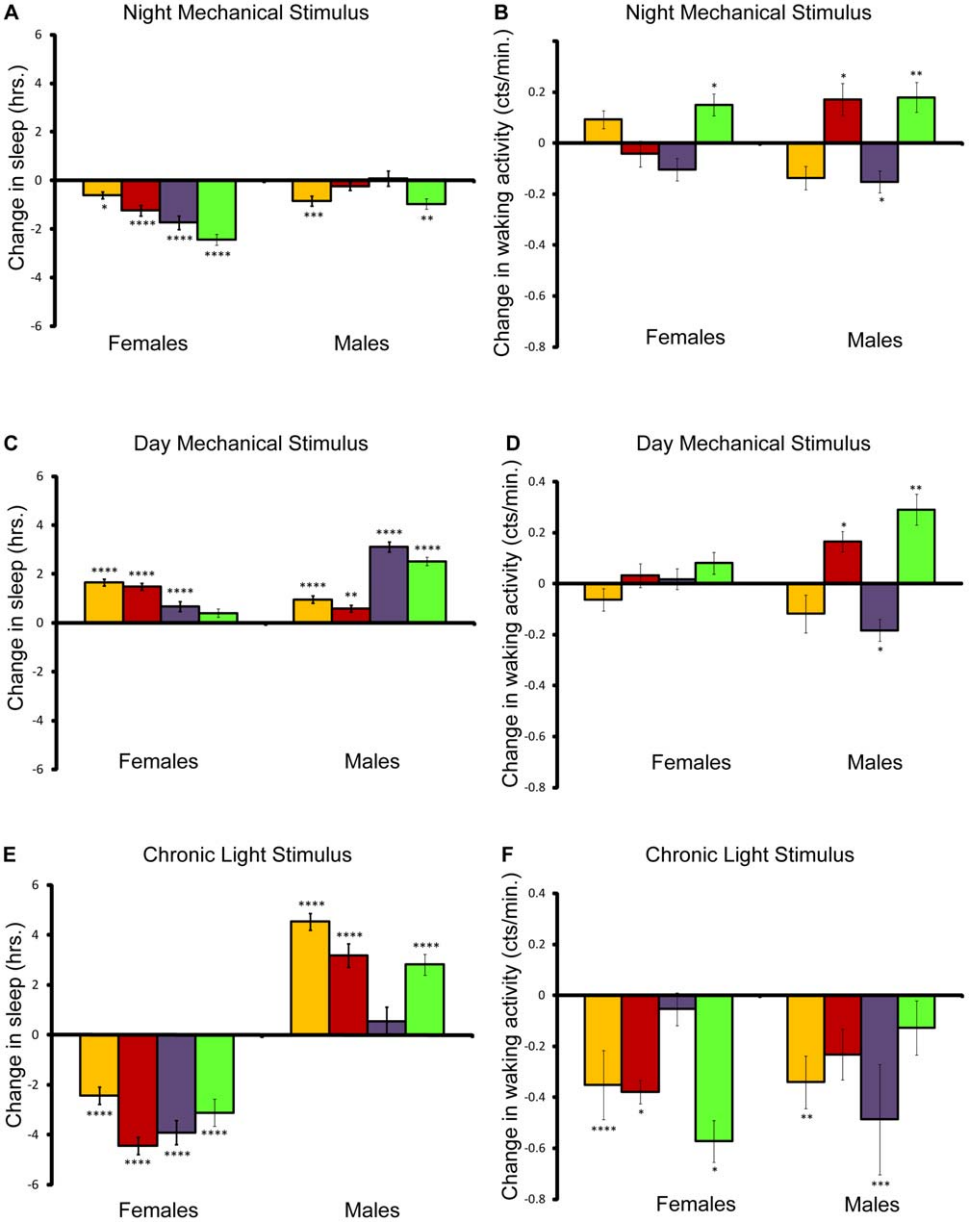
Effect of mechanical and light stimuli on sleep and waking activity. Amounts shown relative to age/sex-matched control. *P* values reflect significance relative to controls for each line and sex. Yellow bars, *w* ^1118^; *Canton-S*; red bars, *Canton-S*; purple bars, *Oregon*, and green bars, 22-2. ****P<.0001; ***P<.001; **P<.01; *P<.05. Error bars represent the standard error of the mean. (A), Change in sleep per day for flies mechanically stimulated at night. (B), Change in waking activity per day for flies mechanically stimulated at night. (C), Change in sleep per day for flies mechanically stimulated during the day. (D), Change in waking activity per day for flies mechanically stimulated during the day. (E), Change in sleep per day for flies stimulated by light. (F), Change in waking activity per day for flies stimulated by light.

To assess the impact of long-term, partial sleep deprivation on energy stores, we deprived flies of four different wild type strains of sleep for seven days using either a mechanical or a light stimulus according to the protocol in Figure 1. Each strain was compared to its corresponding age- and environment-matched controls in four independent experiments (see Methods). The results of the statistical analysis for all conditions are presented in Table S1. The data reveal that the brief two-hour period of mechanical stimulation in the late night produced a net sleep loss per day in both males and females (Figure 2A). Females from all four strains lost sleep each day as compared to controls; females lost as little as 7.65% (*Canton-S*) to as much as 21% (22-2) of sleep per day on average. Males were less affected by the mechanical stimulus, losing 7.44% (22-2) of sleep at most per day on average. To determine if our experimental manipulation induced hyperactivity, we compared the waking activity (average activity counts for the time spent awake) for each line/sex to controls (Figure 2B). With the exception of line 22-2, waking activity did not change significantly in females. Waking activity did change significantly in males of three of the four lines, increasing significantly in *Canton-S* and 22-2 males and decreasing in *Oregon* males.

In contrast to flies mechanically stimulated at night, both male and female flies that were stimulated in the same manner during the day, when they would normally be active, showed increased sleep relative to controls (Figure 2C). Interestingly, alterations in waking activity relative to controls in flies mechanically stimulated during the day were similar to those in flies mechanically stimulated at night (Figure 2D).

We also attempted to deprive flies of sleep by turning on the lights during the night. We exposed flies to eight additional hours of light for seven days and compared their behavior to age-matched controls in two separate experimental blocks. Female flies lost considerable amounts of sleep as compared with their respective controls when stimulated by light during the night, from 25.8% to 41.0% per day on average (Figure 2E). Previous studies have shown that male flies tend to sleep more during daylight hours than females, suggesting that males would be less responsive to the light stimulus [23]. Indeed, we observed a net increase in male sleep each day (Figure 2E). In general, waking activity decreased in both males and females stimulated by light (Figure 2F), despite the fact that the females lost sleep, while the males did not.

**Figure 3:**
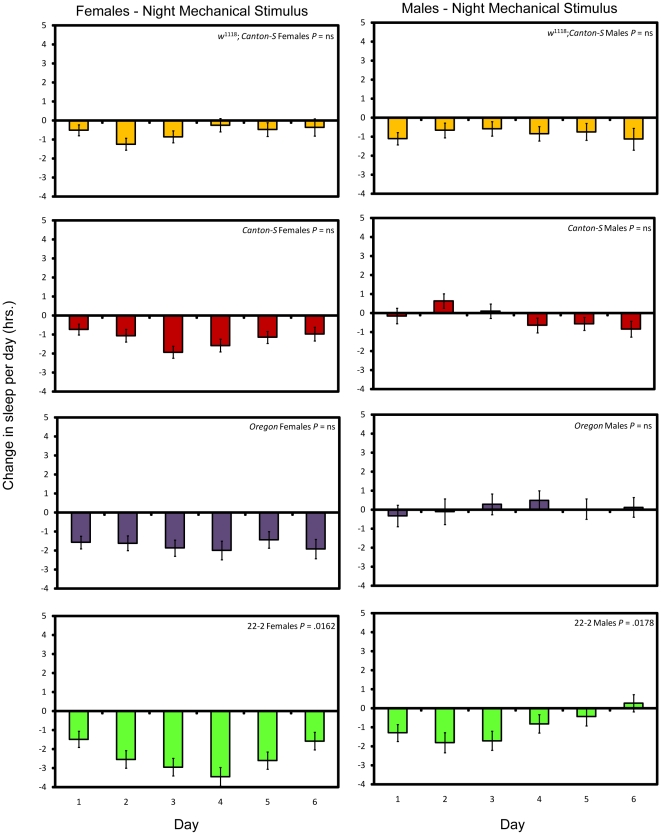
Sleep change per day after long-term mechanical stimulation at night. Yellow bars, *w* ^1118^; *Canton-S*; red bars, *Canton-S*; purple bars, *Oregon*, and green bars, 22-2. *P* values given reflect the significance of the effect of day. Error bars represent the standard error of the mean.

**Figure 4:**
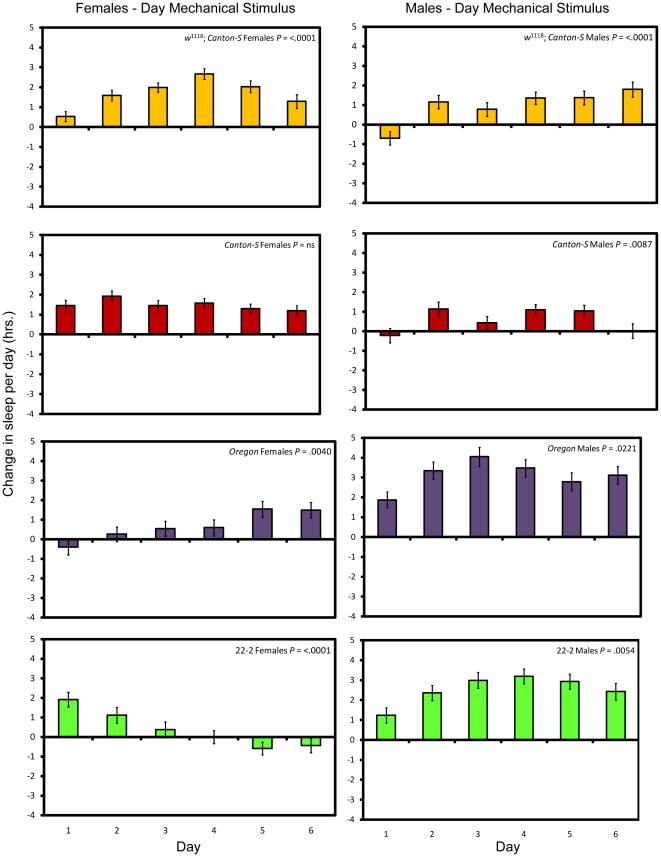
Sleep change per day after long-term mechanical stimulation during the day. Yellow bars, *w* ^1118^; *Canton-S*; red bars, *Canton-S*; purple bars, *Oregon*, and green bars, 22-2. *P* values given reflect the significance of the effect of day. Error bars represent the standard error of the mean.

Flies did not fully compensate for sleep loss caused by the mechanical stimulus applied at night by sleeping more; with the exception of introgression line 22-2, sleep lost on the first day was not significantly different from sleep lost on successive days (Figure 3; see Methods and Table S2). Thus in general the flies do not adapt to the mechanical stimulus when it is applied at night. Flies mechanically stimulated during the day, on the other hand, have a more variable sleep pattern across days, consisting of both increases and decreases in daily sleep (Figure 4 and Table S2). Long-term mechanical stimulation, therefore, produces a net sleep loss only when applied at a time when flies would normally be asleep.

**Figure 5:**
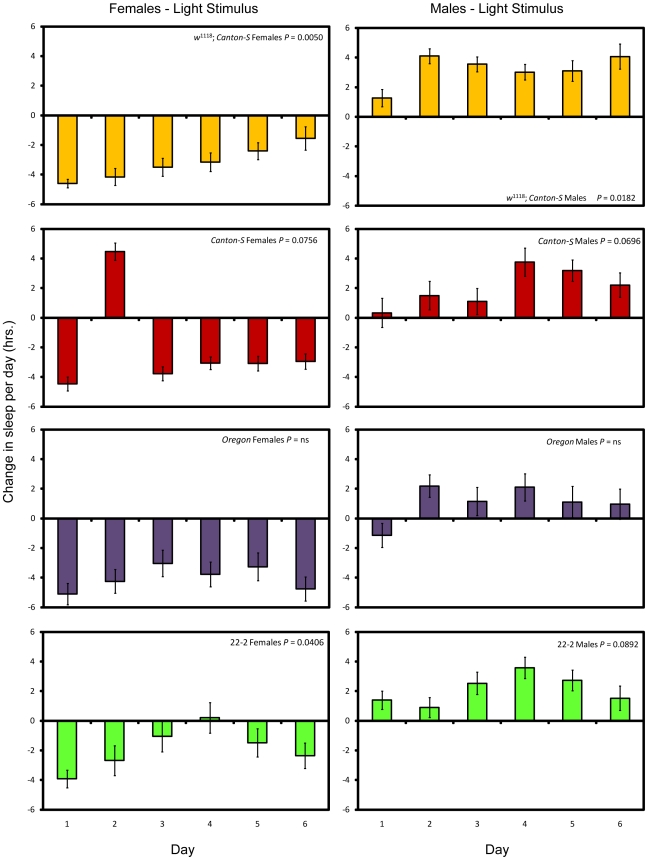
Sleep change per day after long-term light stimulation. Yellow bars, *w* ^1118^; *Canton-S*; red bars, *Canton-S*; purple bars, *Oregon*, and green bars, 22-2. *P* values given reflect the significance of the effect of day. Error bars represent the standard error of the mean.

When stimulated by light, males lost sleep overnight but compensated for the loss during the day. With the exception of *w* ^1118^; *Canton-S* females, females did not compensate for the loss of sleep due to the light stimulus (Figure 5; see Methods and Table S2). Sleep in *w* ^1118^; *Canton-S* females was progressively increased over time; thus, it would appear that females of this line compensated for sleep loss. We therefore successfully deprived female flies of sleep over a long period via two independent methods; males were deprived of sleep using the mechanical stimulus but not the light stimulus.

### Effect of chronic partial sleep deprivation on energy stores

**Figure 6:**
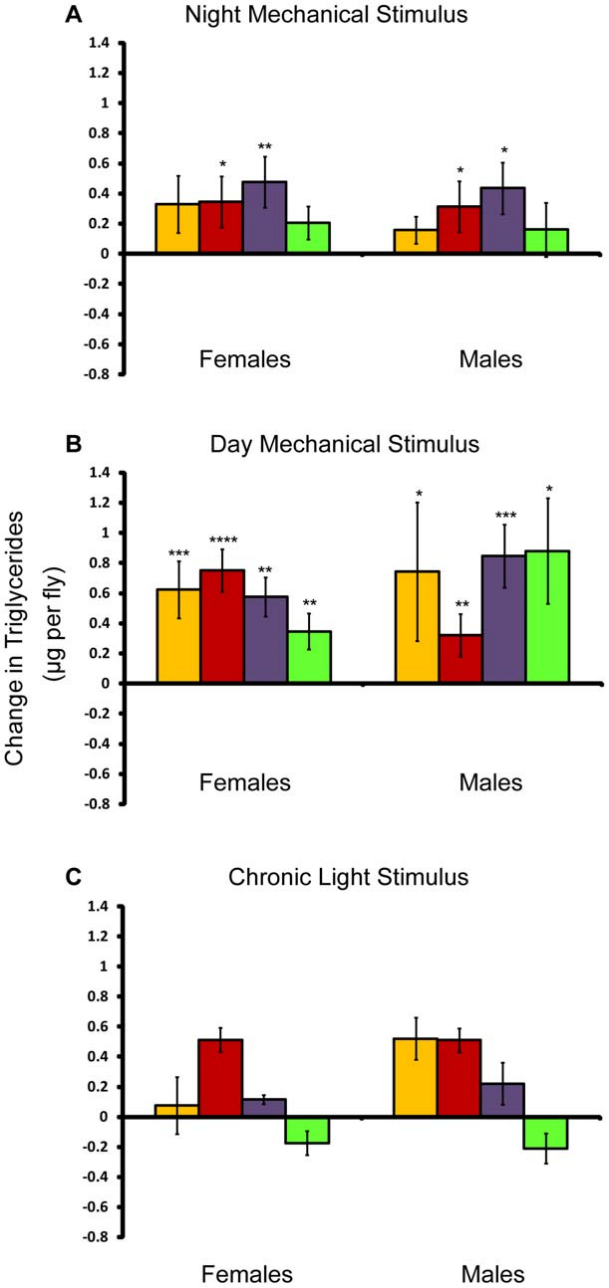
Effect of the mechanical and light stimulus on triglycerides. Amounts are shown relative to age/sex-matched control. *P* values reflect significance relative to controls for each line and sex. ****P<.0001; ***P<.001; **P<.01; *P<.05. Error bars represent the standard error of the mean. Yellow bars, *w* ^1118^; *Canton-S*; red bars, *Canton-S*; purple bars, *Oregon*, and green bars, 22-2. Change in whole-body triglycerides in μg per fly for (A) flies mechanically stimulated at night, (B) flies mechanically stimulated during the day, and (C) flies stimulated by light.

We measured whole-body triglyceride levels in flies after they had been sleep deprived for seven days and compared them to age- and environment-matched controls. Triglyceride stores increased in all mechanically stimulated flies, whether the stimulation occurred during the day or at night (Figure 6A and 6B). Triglycerides were also altered after light stimulation (Figure 6C), but the effects consisted of non-significant decreases as well as increases. In particular, we observed no significant changes in triglycerides in females, who were deprived of sleep by the light stimulus. Thus, long-term mechanical sleep deprivation at night reduced sleep and increased triglyceride stores, while deprivation using the light stimulus reduced sleep in females and did not significantly affect triglycerides. However, mechanically stimulating flies during the day, which increased sleep, also increased triglyceride stores. These findings indicate that a loss of sleep was not the reason for the increase in triglycerides in flies deprived at night with the mechanical stimulus.

**Figure 7:**
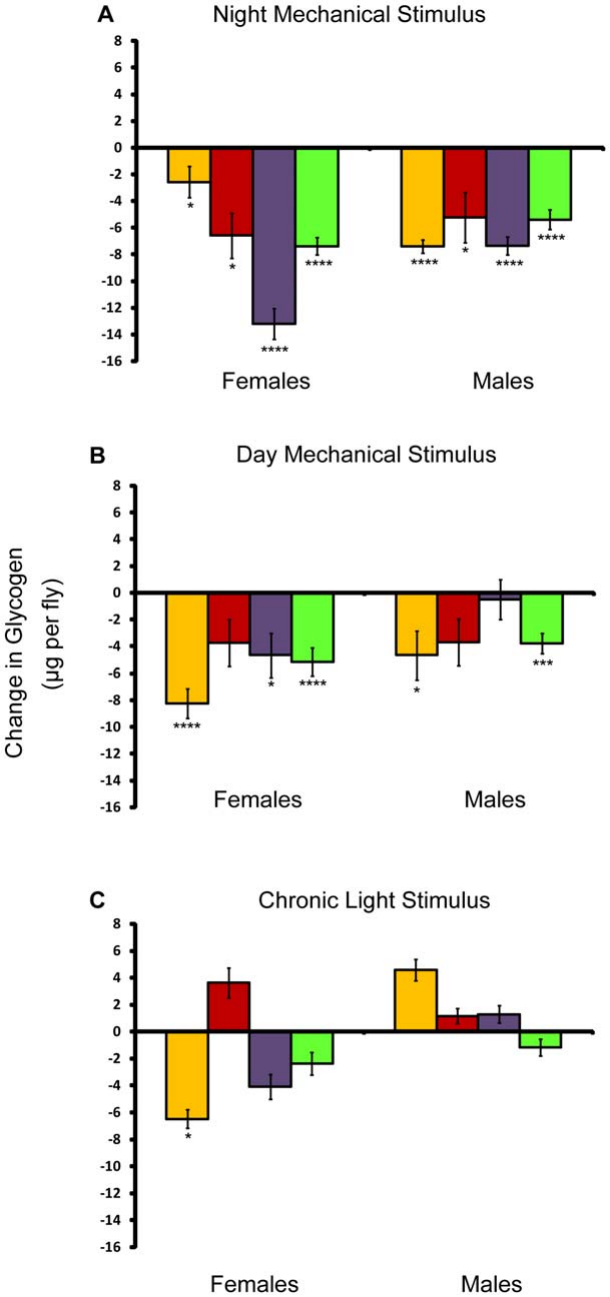
Effect of the mechanical and light stimulus on glycogen. Amounts are shown relative to age/sex-matched control. *P* values reflect significance relative to controls for each line and sex. ****P<.0001; ***P<.001; **P<.01; *P<.05. Error bars represent the standard error of the mean. Yellow bars, *w* ^1118^; *Canton-S*; red bars, *Canton-S*; purple bars, *Oregon*, and green bars, 22-2. Change in whole-body glycogen in μg per fly for (A) flies mechanically stimulated at night, (B) flies mechanically stimulated during the day, and (C) flies stimulated by light.

We also assessed the effect of sleep deprivation on whole-body glycogen stores. Glycogen was measured after seven days of sleep deprivation and compared to age- and environment-matched controls. Whole-body glycogen stores were reduced considerably in response to long-term mechanical stimulation, whether the stimulation occurred during the day or at night (Figure 7A and 7B). However, light stimulation had little effect on glycogen stores with the exception of a large decrease seen in *w* ^1118^; *Canton-S* females (Figure 7C). Neither males, who slept longer in the presence of the light stimulus, nor females, who lost sleep, had significant differences in glycogen stores when compared to the control. Thus, as for the triglycerides, the decrease in glycogen by the mechanical stimulus does not appear to be due to a loss of sleep. Alternatively, since light can have many profound effects on an organism, other factors may have dampened the effects produced by light-induced sleep loss in females (see below).

### Effect of circadian clock shifts on energy stores

**Figure 8:**
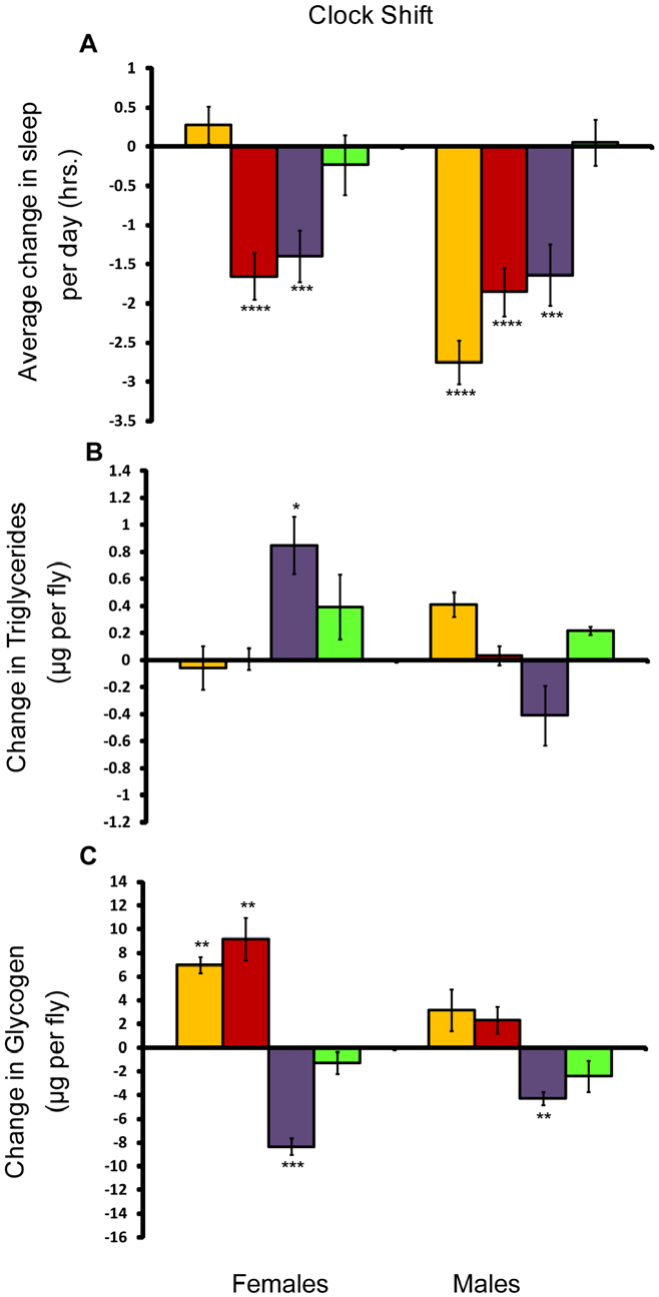
Effect of the clock shift on sleep, triglycerides, and glycogen. Amounts shown relative to age/sex-matched control. *P* values reflect significance relative to controls for each line and sex. ****P<.0001; ***P<.001; **P<.01; *P<.05. Error bars represent the standard error of the mean. Yellow bars, *w* ^1118^; *Canton-S*; red bars, *Canton-S*; purple bars, *Oregon*, and green bars, 22-2. (A) Average change in sleep per day for shifted flies; (B) Change in whole-body triglycerides in μg per fly; (C) Change in whole-body glycogen in μg per fly.

Sleep behavior is intertwined with circadian rhythms. While the mechanical stimulation protocol does not alter the circadian clock [17], the seven-day chronic light stimulus protocol may have had an effect [24]. Since it is possible that effects on the clock countered effects on sleep, we measured the effect of shifting the clock multiple times over a 12 day period on energy stores (see Methods and Figure 1). We designed the shifts so that at the end of the experiment, the flies had the same amount of light exposure as their age-matched controls. Although the clock-shift experiment was not intended to deprive flies of sleep, both males and females lost sleep (Figure 8A), underscoring the difficulty in uncoupling sleep from the circadian clock.

We saw little effect of the clock shift on triglycerides; as Figure 8B shows, only *Oregon* females exhibited significant changes over their control, increasing triglyceride stores. The effect on glycogen was more widespread, with significant increases and decreases, indicating a sex- and line-specific response to the clock shift (Figure 8C). Since the pattern was different across line, we infer that shifting the clock does not have consistent, predictable effects on glycogen stores. In addition, since the clock shift produced sleep loss, these data support the conclusion above that long-term sleep loss is not associated with consistent changes in glycogen or triglycerides.

### Effect of acute sleep deprivation on energy stores

**Figure 9:**
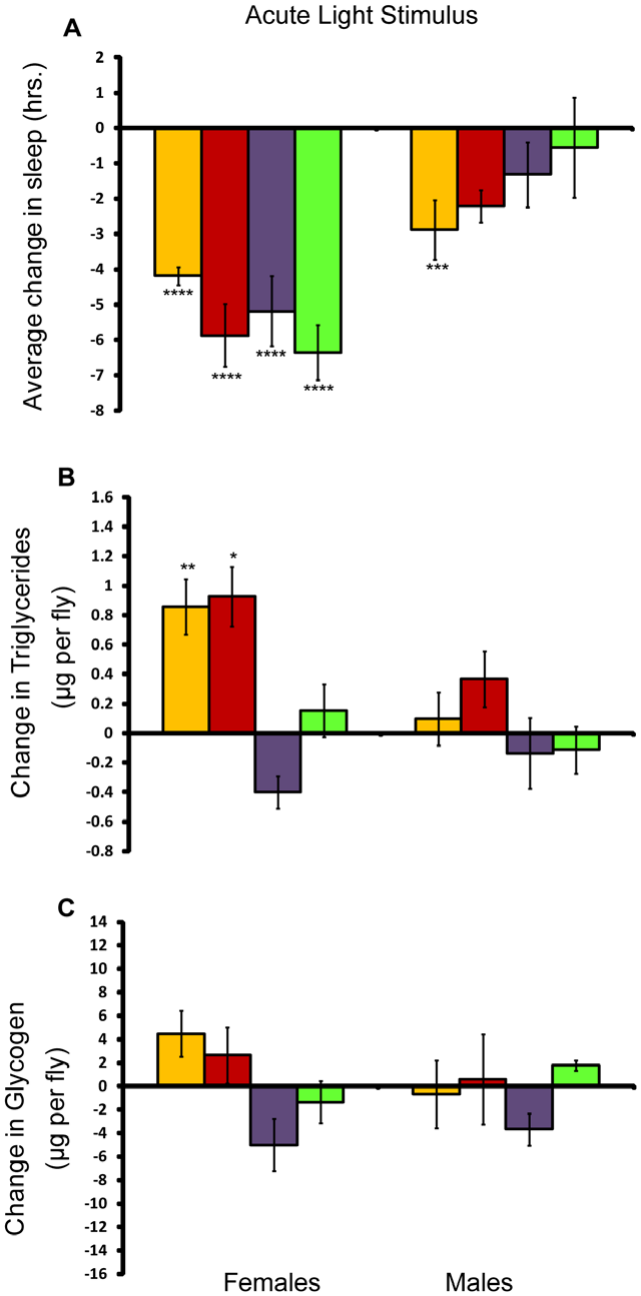
Effect of an acute light stimulus on sleep, triglycerides, and glycogen. Amounts shown relative to age/sex-matched control. *P* values reflect significance relative to controls for each line and sex. Error bars represent the standard error of the mean. Yellow bars, *w* ^1118^; *Canton-S*; red bars, *Canton-S*; purple bars, *Oregon*, and green bars, 22-2. ****P<.0001; ***P<.001; **P<.01; *P<.05. (A) Change in sleep after a single day of light stimulation; (B) Change in whole-body triglycerides in μg per fly; (C) Change in whole-body glycogen in μg per fly.

Our results indicate that chronic light-induced sleep deprivation does not affect energy stores. In addition to potentially affecting the circadian clock as mentioned above, the flies may have acclimated to the light stimulation over time. Post-hoc Tukey analysis revealed that the difference in sleep in most of the light-stimulated flies and their respective controls changed significantly (P<.05) over the course of the seven-day experiment (see Figure 5). *w* ^1118^; *Canton-S* females in particular appeared to adapt to the light stimulus over time, increasing sleep each day. We therefore tested the effect of a single day of the light stimulus protocol on energy stores. Overnight sleep was greatly reduced in females exposed to the acute light stimulus (Figure 9A); for example, 22-2 females lost 5.87 hours of sleep. Unlike in the chronic light stimulus protocol, males did not have the opportunity to compensate for lost sleep. Thus, they also exhibited sleep loss when the light stimulus was applied overnight although the loss of sleep was only statistically significant in *w* ^1118^; *Canton-S* males (2.88 hours). Triglycerides in males were virtually unaffected (Figure 9B). Despite losing significant quantities of sleep, *Oregon* and 22-2 females also did not exhibit significant changes in triglycerides. However triglycerides in *w* ^1118^; *Canton-S* and *Canton-S* females had significant increases. Glycogen levels did not change significantly in response to the acute light stimulus for any line or sex (Figure 9C). Thus, as with the long-term sleep deprivation using the light stimulus, acute sleep loss did not significantly impact glycogen stores. The response of triglycerides to acute sleep deprivation was both sex-and line-dependent.

## Discussion

We deprived flies of sleep using two methods: a mechanical stimulus and a light stimulus. The mechanical stimulus produced sleep loss when applied at night, but not during the day. When chronically sleep-restricted during the night, flies only partly compensated for the sleep loss, consistent with studies in both rodents and humans [25], [26]. When exposed to the same mechanical stimulation during the day, flies slept more than controls. Yet energy stores in both groups of flies displayed a pattern that was consistent across lines and sexes: glycogen levels decreased, while triglycerides increased. These data suggest that the changes in energy stores that we observed are not due to chronic partial sleep loss. Supporting evidence is provided by our findings for flies deprived of sleep using the light stimulus. While not effective at chronically depriving males of sleep, the additional light produced greater sleep loss in females than the mechanical stimulus. However, the pattern of decreased glycogen and increased triglycerides seen after mechanical stimulation was not seen in females stimulated by light. Instead, triglycerides were not significantly altered as compared to the controls, and glycogen was significantly reduced in only one line (*w* ^1118^; *Canton-S*). Taken together, the data indicate that chronic partial sleep loss per se does not impact energy stores. Furthermore, our circadian clock shift experiment was not intended to deprive flies of sleep, but they did lose sleep. Despite the sleep loss, the clock shift experiment had little effect on triglycerides, reinforcing the conclusion that changes in triglycerides were a result of factors other than sleep loss. The effect of the clock shift on glycogen varied among sexes and lines. Since the clock shift was not de-coupled from the sleep loss, two conclusions are possible: alterations in the molecular circadian clock affected glycogen stores, or the sleep loss in combination with changes in the molecular circadian clock affected glycogen. Recent evidence that the fly circadian clock controls feeding behavior argues for the former conclusion. Peripheral clocks in the fat body (analogous to the mammalian liver) inhibited nighttime feeding [27] and disruption of this peripheral clock reduced glycogen levels in the fat body, an effect which was opposed by disrupting the circadian clock in neuronal cells [27]. Although the current study examined whole-body glycogen, these findings nevertheless imply that the circadian clock can affect glycogen stores in the fly.

It is not clear why the mechanically stimulated flies exhibited increases in triglycerides while having a reduction in glycogen stores. To keep the flies awake, the mechanical stimulus physically perturbs the flies, which may result in an increase in their activity. For example, loss of glycogen has been observed in both the heads and bodies of female flies after sleep deprivation by hand tapping, whether the flies were stimulated during their normal sleep period or during their active period [28]. One possibility is that the large reduction in whole-body glycogen that we observed could be due to increases in locomotor activity elicited by the mechanical stimulus. However, if changes glycogen or triglycerides were solely mediated by changes in total activity counts resulting from altered sleep, then one would always observe the same relationship between these nutrients and sleep time. The observations herein suggest that the total amount of activity is not the sole determinant of glycogen or triglyceride levels.

Furthermore, we did not observe a consistent pattern between changes in energy stores and changes in waking activity. Nor was waking activity uniformly increased with the application of the mechanical stimulus, underscoring the previously observed lack of correlation between sleep time and waking activity [29], [30].

We suggest that the increase in triglycerides we observed after mechanical stimulation may be induced as part of the stress response. A number of stress response pathways are conserved between mammals and flies [31], [32]. Recent studies have shown that stress pathway molecules such as c-Jun N-terminal kinase, which is conserved in flies, influence insulin signaling and fat storage [33]. Flies deprived of sleep using the mechanical stimulus have increased expression of genes involved in stress response pathways, including genes involved in the inflammatory, oxidative stress, and unfolded protein responses [34], which may account for the increase in triglycerides we observed. Thus, stress is more likely the stronger influence on triglyceride level when flies are mechanically stimulated. How stress might alter triglyceride level, whether through changes in physiology or behaviors such as feeding, remains to be determined.

When human sleep was restricted to four hours for six days, impaired carbohydrate metabolism and endocrine function were observed [35], leading the authors to suggest that long-term sleep loss might produce metabolic changes increasing the likelihood of obesity and type 2 diabetes [35], [36]. In this experiment, we did not observe changes in glycogen or triglyceride stores that would suggest similar metabolic changes take place with chronic partial sleep loss in flies. However, in a parallel study, we observed a genetic link between endogenous sleep and energy stores [20]. Based upon the metabolic effects of stress discussed above, we suggest that stress may contribute to the effects of short sleep on metabolism.

## Materials and Methods

### Drosophila stocks

Wild-type fly lines were used to assay the effect of sleep deprivation on energy stores. Common laboratory strains *Oregon-R* and *Canton-S* were used, as well as an isogenized *w* ^1118^; *Canton-S* strain created as part of the Berkeley Drosophila Genome Disruption Project [37]. We also assayed a recombinant inbred line derived from *Oregon-R* and the Russian *2b* strain, 22-2 [38].

Flies were reared and maintained on standard medium in a 25°C, 12-hour light/dark cycle incubator. All rearing cultures were adult-density controlled at five males and five females per vial. For all assays, adult virgins were collected and maintained at 30 flies to a single-sex vial until the time of assay to mitigate the effects of social enrichment on sleep [39] and to give equal access to the food source. Flies had access to food at all times.

### Sleep behavior monitoring

For all manipulations (mechanical stimulus, light stimulus, or clock shift), we monitored sleep and activity using the Drosophila Activity Monitoring System (Trikinetics, Waltham, MA) [40]. Sleep and activity were quantified using an in-house C^++^ program that calculated hours of sleep, numbers of sleep bouts, average bout length, and activity counts per waking minute (waking activity). Sleep was defined as any period five minutes or longer without an activity count [17], [18], [23], [40].

### Sleep deprivation using mechanical stimulus

We subjected wild-type flies to long-term sleep deprivation using a mechanical stimulus as described [23]. We deprived flies of sleep for two hours each day over a seven-day period. This protocol resulted in a 10–25% loss of sleep per night, based on preliminary data. We divided flies into three treatment groups: mechanically stimulated at night (Night Mechanical Stimulus), mechanically stimulated during the day (Day Mechanical Stimulus), and un-stimulated controls (see Figure 1). Sixty-four flies of each sex per line were assayed per treatment group in four experimental blocks. At the end of the sleep deprivation period, we measured energy storage parameters as described below.

### Sleep deprivation using light stimulus

To discriminate between the effects of sleep loss and effects that were solely due to the mechanical stimulus, we deprived flies of sleep using light (see Figure 1). The Chronic Light Stimulus protocol consisted of exposing flies to eight hours of additional light during their normal 12-hour dark cycle in the following pattern: two hours dark, eight hours light, and two hours dark. After seven days, we harvested flies at the beginning of the normal light cycle and assayed them for energy storage parameters. Flies subjected to the light stimulus were compared to age-matched controls subjected to the usual 12 hr light: 12 hr dark cycle. We assayed 32 flies of each sex per line in each treatment group in two experimental blocks. To account for possible adaptation to the light stimulus over time, we subjected flies to a single night of sleep deprivation using light. The Acute Light Stimulus protocol was the same as the Chronic Light Stimulus protocol, except that flies were harvested after a single day of exposure to additional light.

### Clock shift assay

We examined the impact of a long-term random shift in the fly circadian clock on energy stores. Importantly, the Clock Shift assay was designed to give flies the same amount of light as control flies on a normal 12-hour light∶dark schedule. This methodology enabled us to distinguish between potential effects on energy stores due to manipulation of the circadian clock and to different amounts of light. We shifted the clock for 12 days (see Figure 1). We tested 32 flies of each sex per line in each treatment group in two experimental blocks. Note that at the end of the experiment, both experimental flies and their age-matched controls had the same circadian light∶dark cycle. All flies were harvested at the same circadian time and assayed for energy stores.

### Measurement of energy stores

For homogenization, all flies were collected at the beginning of their lights-on period (see Figure 1). Flies were weighed in groups of ten; whole bodies (including the head) were homogenized on ice in 0.01M KH_2_PO_4_, 1 mM EDTA pH 7.4 buffer as described [41]. We used 25 µl of homogenizing buffer per fly. Homogenates were immediately used to measure whole-body protein, glycogen, and triglycerides. Each colorimetric assay was read using a Perkin-Elmer V^3^ plate reader (Waltham, MA). Bradford's method was used to determine the protein in μg per fly [42]; BSA was used for the protein standard curve. We measured total glycogen in μg per fly as described [41]. Briefly, glycogen from the homogenates was broken down into glucose by adding 0.1 U/ml amyloglucosidase enzyme slurry (Sigma) to 1.5 µl samples of homogenate in a 96-well plate. Total glucose was then determined using the PGO Enzymes Kit (Sigma) [41]. Free glucose is estimated at less than 5% of the amount of glycogen stored [41]; thus, this measure is effectively the amount of whole-body glycogen. Glucose concentrations were determined using a glucose standard curve run on the same plate. Known concentrations of glycogen were used as standards to assess the expected recovery of glycogen [28]; we repeated the measurements if less than 95% of the glycogen standard was recovered. True serum triglycerides in μg per fly were determined using an enzymatic assay kit (Serum Triglyceride Determination Kit, Sigma-Aldrich, St. Louis, MO) [43]. Homogenates were then stored at −80°C, and measurements were repeated the next day. Two separate biological replicates were assayed for the clock shift assay and the assays using light to deprive flies of sleep; four separate replicates were performed for the mechanical sleep deprivation assays.

### Statistical analysis

We used the following ANOVA model to assess the changes in sleep and energy storage phenotypes after sleep deprivation: y = μ+L+S+T+L×S+L×T+S×T+L×S×T+E, where L is the line, S is sex, T is the treatment (control or sleep-deprived), and E is the within-tube environmental variance. Comparisons among mechanically-stimulated flies using this model were highly significant (P<0.05), indicating differences among sexes and lines as well as among treatments. We therefore determined the effect of each treatment on sleep for each line/sex combination separately using the reduced model y = μ+T+E, where T is the treatment (control, deprived, etc.) and E is the environmental variance within treatments. Note that initially we used body weight as a covariate in the energy storage analyses; however, we found that there were no significant differences among treatments or among lines for body weight. We thus dropped the weight term from our reduced analysis. We performed a post-hoc Tukey analysis for each parameter in order to rank differences between treatments.

We performed an additional analysis on the flies deprived of sleep using the mechanical or the light stimulus to see if the flies were adapting to either stimulus over time. We subtracted the average 24-hour sleep for each control line/sex per day from the respective mechanical- or light-stimulated group. We then analyzed these differences in an ANOVA model: y = μ+D+E, where D is day and E is the variance among individuals. We performed a post-hoc Tukey analysis on this data, which ranked the difference in sleep observed between the control and the light-stimulated flies by day. Flies were considered to be compensating for the sleep-depriving stimulus if they met the following two criteria. First, ANOVA results had to reveal a statistically significant effect of day on the change in sleep (P<.05). Secondly, the post-hoc Tukey ranking had to indicate that flies had increased sleep for each successive day.

## Supporting Information

Table S1 Analyses of variance of sleep traits.
(0.22 MB DOC)

Table S2 Post-hoc Tukey analysis to detect adaptation to mechanical and light stimuli.
(0.15 MB DOC)
