# Supplementary material for: Energy Stores Are Not Altered by Long-Term Partial Sleep Deprivation in Drosophila melanogaster
Source: PLoS One. 2009 Jul 10;4(7):e6211. doi: 10.1371/journal.pone.0006211 (PMC2703806; doi:10.1371/journal.pone.0006211)
Supplement: Table S1 — Analyses of variance of sleep traits. (0.22 MB DOC) [file pone.0006211.s001.doc]

**Table S1.** Analyses of variance of sleep traits.

| Analysis | Trait | Source | d.f. | MS | *F* | *P* |
| --- | --- | --- | --- | --- | --- | --- |
| Mechanical | Sleep (hr) | Sex | 1 | 3002.91 | 401.52 | <0.0001 |
| sleep deprivation |  | Line | 3 | 364.84 | 48.78 | <0.0001 |
| (both day and night) |  | Treatment | 2 | 760.63 | 101.70 | <0.0001 |
|  |  | Line × Sex | 3 | 798.49 | 106.77 | <0.0001 |
|  |  | Line × Treatment | 6 | 17.52 | 2.34 | 0.0296 |
|  |  | Sex × Treatment | 2 | 36.96 | 4.94 | 0.0073 |
|  |  | Line × Sex × Treatment | 6 | 31.04 | 4.15 | 0.0004 |
|  |  | Error | 1374 | 7.47 | -- | -- |
|  |  |  |  |  |  |  |
|  | Waking Activity | Sex | 1 | 53.59 | 5.27 | 0.0219 |
|  | (Counts/min.) | Line | 3 | 25.82 | 2.54 | 0.0551 |
|  |  | Treatment | 2 | 11.39 | 1.12 | 0.3265 |
|  |  | Line × Sex | 3 | 13.84 | 1.36 | 0.2535 |
|  |  | Line × Treatment | 6 | 11.88 | 1.17 | 0.3208 |
|  |  | Sex × Treatment | 2 | 9.43 | 0.93 | 0.3959 |
|  |  | Line × Sex × Treatment | 6 | 8.15 | 0.80 | 0.5683 |
|  |  | Error | 1374 | 14.77 | -- | -- |
|  |  |  |  |  |  |  |
|  | Triglycerides | Sex | 1 | 35.59 | 49.28 | <0.0001 |
|  | (µg per fly) | Line | 3 | 43.31 | 59.97 | <0.0001 |
|  |  | Treatment | 2 | 13.00 | 18.00 | <0.0001 |
|  |  | Line × Sex | 3 | 3.44 | 4.77 | 0.0028 |
|  |  | Line × Treatment | 6 | 0.18 | 0.25 | 0.9578 |
|  |  | Sex × Treatment | 2 | 0.32 | 0.44 | 0.6459 |
|  |  | Line × Sex × Treatment | 6 | 0.46 | 0.64 | 0.7011 |
|  |  | Error | 360 | 0.72 | -- | -- |
|  |  |  |  |  |  |  |
|  | Glycogen | Sex | 1 | 2954.43 | 93.53 | <0.0001 |
|  | (µg per fly) | Line | 3 | 3167.36 | 100.28 | <0.0001 |
|  |  | Treatment | 2 | 1541.07 | 48.79 | <0.0001 |
|  |  | Line × Sex | 3 | 226.01 | 7.16 | 0.0001 |
|  |  | Line × Treatment | 6 | 132.00 | 4.18 | 0.0004 |
|  |  | Sex × Treatment | 2 | 52.06 | 1.65 | 0.1938 |
|  |  | Line × Sex × Treatment | 6 | 61.67 | 1.95 | 0.0717 |
|  |  | Error | 360 | 31.58 | -- | -- |
|  |  |  |  |  |  |  |
| Sleep deprivation | Sleep (hr) | Sex | 1 | 2184.66 | 256.26 | <0.0001 |
| using light |  | Line | 3 | 90.88 | 10.66 | <0.0001 |
|  |  | Treatment | 1 | 3.86 | 0.45 | 0.5012 |
|  |  | Line × Sex | 3 | 279.06 | 32.73 | <0.0001 |
|  |  | Line × Treatment | 3 | 18.65 | 2.19 | 0.0889 |
|  |  | Sex × Treatment | 1 | 785.89 | 92.19 | <0.0001 |
|  |  | Line × Sex × Treatment | 3 | 7.77 | 0.91 | 0.4353 |
|  |  | Error | 431 | 8.53 | -- | -- |
|  |  |  |  |  |  |  |
|  | Waking Activity | Sex | 1 | 11.17 | 26.13 | <0.0001 |
|  | (Counts/min.) | Line | 3 | 3.04 | 7.10 | 0.0001 |
|  |  | Treatment | 1 | 11.05 | 25.83 | <0.0001 |
|  |  | Line × Sex | 3 | 0.53 | 1.23 | 0.2988 |
|  |  | Line × Treatment | 3 | 0.11 | 0.25 | 0.8621 |
|  |  | Sex × Treatment | 1 | 0.15 | 0.35 | 0.5550 |
|  |  | Line × Sex × Treatment | 3 | 0.94 | 2.19 | 0.0887 |
|  |  | Error | 431 | 0.43 | -- | -- |
|  |  |  |  |  |  |  |
|  | Triglycerides | Sex | 1 | 0.18 | 0.08 | 0.7844 |
|  | (µg per fly) | Line | 3 | 6.83 | 2.88 | 0.0411 |
|  |  | Treatment | 1 | 1.52 | 0.64 | 0.4262 |
|  |  | Line × Sex | 3 | 1.35 | 0.57 | 0.6354 |
|  |  | Line × Treatment | 3 | 0.68 | 0.29 | 0.8344 |
|  |  | Sex × Treatment | 1 | 0.20 | 0.09 | 0.7713 |
|  |  | Line × Sex × Treatment | 3 | 0.16 | 0.07 | 0.9780 |
|  |  | Error | 78 | 2.37 | -- | -- |
|  |  |  |  |  |  |  |
|  | Glycogen | Sex | 1 | 511.14 | 9.40 | 0.0030 |
|  | (µg per fly) | Line | 3 | 693.54 | 12.76 | <0.0001 |
|  |  | Treatment | 1 | 4.36 | 0.08 | 0.7778 |
|  |  | Line × Sex | 3 | 264.07 | 4.86 | 0.0037 |
|  |  | Line × Treatment | 3 | 21.97 | 0.40 | 0.7505 |
|  |  | Sex × Treatment | 1 | 86.74 | 1.60 | 0.2102 |
|  |  | Line × Sex × Treatment | 3 | 50.32 | 0.93 | 0.4305 |
|  |  | Error | 80 | 54.37 | -- | -- |
|  |  |  |  |  |  |  |
| Clock shift | Sleep (hr) | Sex | 1 | 363.23 | 55.11 | <0.0001 |
|  |  | Line | 3 | 137.81 | 20.91 | <0.0001 |
|  |  | Treatment | 1 | 44.98 | 6.82 | 0.0096 |
|  |  | Line × Sex | 3 | 145.41 | 22.06 | <0.0001 |
|  |  | Line × Treatment | 3 | 12.74 | 1.93 | 0.1252 |
|  |  | Sex × Treatment | 1 | 5.48 | 0.83 | 0.3626 |
|  |  | Line × Sex × Treatment | 3 | 5.86 | 0.89 | 0.4475 |
|  |  | Error | 218 | 6.59 | -- | -- |
|  |  |  |  |  |  |  |
|  | Waking Activity | Sex | 1 | 11.30 | 46.60 | <0.0001 |
|  | (Counts/min.) | Line | 3 | 3.94 | 16.25 | <0.0001 |
|  |  | Treatment | 1 | 0.05 | 0.21 | 0.6440 |
|  |  | Line × Sex | 3 | 0.70 | 2.89 | 0.0363 |
|  |  | Line × Treatment | 3 | 0.61 | 2.51 | 0.593 |
|  |  | Sex × Treatment | 1 | 0.05 | 0.20 | 0.6546 |
|  |  | Line × Sex × Treatment | 3 | 0.32 | 1.31 | 0.2712 |
|  |  | Error | 218 | 0.24 | -- | -- |
|  |  |  |  |  |  |  |
|  | Triglycerides | Sex | 1 | 2.16 | 2.25 | 0.1374 |
|  | (µg per fly) | Line | 3 | 15.87 | 16.54 | <0.0001 |
|  |  | Treatment | 1 | 0.23 | 0.24 | 0.6281 |
|  |  | Line × Sex | 3 | 0.21 | 0.22 | 0.8839 |
|  |  | Line × Treatment | 3 | 1.07 | 1.12 | 0.3462 |
|  |  | Sex × Treatment | 1 | 0.18 | 0.19 | 0.6661 |
|  |  | Line × Sex × Treatment | 3 | 0.23 | 0.24 | 0.8697 |
|  |  | Error | 80 | 0.96 | -- | -- |
|  |  |  |  |  |  |  |
|  | Glycogen | Sex | 1 | 1051.64 | 94.81 | <0.0001 |
|  | (µg per fly) | Line | 3 | 1287.94 | 116.11 | <0.0001 |
|  |  | Treatment | 1 | 1.97 | 0.18 | 0.6742 |
|  |  | Line × Sex | 3 | 215.69 | 19.45 | <0.0001 |
|  |  | Line × Treatment | 3 | 139.29 | 12.56 | <0.0001 |
|  |  | Sex × Treatment | 1 | 22.58 | 2.04 | 0.1576 |
|  |  | Line × Sex × Treatment | 3 | 7.93 | 0.71 | 0.5460 |
|  |  | Error | 80 | 11.09 | -- | -- |
|  |  |  |  |  |  |  |
| Acute sleep | Sleep (hr) | Sex | 1 | 0.42 | 0.12 | 0.7275 |
| deprivation with light |  | Line | 3 | 26.22 | 7.58 | 0.0001 |
|  |  | Treatment | 1 | 405.95 | 117.41 | <0.0001 |
|  |  | Line × Sex | 3 | 8.10 | 2.34 | 0.0768 |
|  |  | Line × Treatment | 3 | 0.85 | 0.24 | 0.8649 |
|  |  | Sex × Treatment | 1 | 106.85 | 30.91 | <0.0001 |
|  |  | Line × Sex × Treatment | 3 | 6.80 | 1.97 | 0.1228 |
|  |  | Error | 112 | 3.46 | -- | -- |
|  |  |  |  |  |  |  |
|  | Waking Activity | Sex | 1 | 1.03 | 8.80 | 0.0037 |
|  | (Counts/min.) | Line | 3 | 0.89 | 7.61 | 0.0001 |
|  |  | Treatment | 1 | 0.88 | 7.53 | 0.0071 |
|  |  | Line × Sex | 3 | 0.22 | 1.87 | 0.1393 |
|  |  | Line × Treatment | 3 | 0.26 | 2.25 | 0.0866 |
|  |  | Sex × Treatment | 1 | 1.30 | 11.08 | 0.0012 |
|  |  | Line × Sex × Treatment | 3 | 0.07 | 0.63 | 0.5967 |
|  |  | Error | 112 | 0.12 | -- | -- |
|  |  |  |  |  |  |  |
|  | Triglycerides | Sex | 1 | 27.04 | 12.46 | 0.0006 |
|  | (µg per fly) | Line | 3 | 15.31 | 7.05 | 0.0002 |
|  |  | Treatment | 1 | 1.22 | 0.56 | 0.4555 |
|  |  | Line × Sex | 3 | 2.35 | 1.08 | 0.3587 |
|  |  | Line × Treatment | 3 | 1.29 | 0.60 | 0.6186 |
|  |  | Sex × Treatment | 1 | 1.14 | 0.53 | 0.4696 |
|  |  | Line × Sex × Treatment | 3 | 0.52 | 0.24 | 0.8662 |
|  |  | Error | 112 | 2.17 | -- | -- |
|  |  |  |  |  |  |  |
|  | Glycogen | Sex | 1 | 3999.89 | 104.63 | <0.0001 |
|  | (µg per fly) | Line | 3 | 1342.34 | 35.11 | <0.0001 |
|  |  | Treatment | 1 | 0.68 | 0.02 | 0.8941 |
|  |  | Line × Sex | 3 | 67.76 | 1.77 | 0.1565 |
|  |  | Line × Treatment | 3 | 66.12 | 1.73 | 0.1650 |
|  |  | Sex × Treatment | 1 | 3.77 | 0.10 | 0.7538 |
|  |  | Line × Sex × Treatment | 3 | 26.74 | 0.70 | 0.5542 |
|  |  | Error | 112 | 38.23 | -- | -- |
|  |  |  |  |  |  |  |

d.f., degrees of freedom; MS, mean squares; *P*, *P* value.
