# Supplementary material for: Energy Stores Are Not Altered by Long-Term Partial Sleep Deprivation in Drosophila melanogaster
Source: PLoS One. 2009 Jul 10;4(7):e6211. doi: 10.1371/journal.pone.0006211 (PMC2703806; doi:10.1371/journal.pone.0006211)
Supplement: Table S2 — Post-hoc Tukey analysis to detect adaptation to mechanical and light stimuli. (0.15 MB DOC) [file pone.0006211.s002.doc]

**Table S2.** Post-hoc Tukey analysis to detect adaptation to mechanical and light stimuli.

| Line | Sex | Stimulus Type | Source | d.f. | MS | *F* | *P* | Minimum Significant Difference (hrs.) | Order of Days |
| --- | --- | --- | --- | --- | --- | --- | --- | --- | --- |
| *w*^1118^; *Canton-S* | Female | Mechanical | Day | 5 | 7.81 | 1.13 | 0.3417 | 1.41 | N/A |
|  |  | stimulus | Error | 336 | 6.88 | -- | -- |  |  |
|  |  | (Night) |  |  |  |  |  |  |  |
|  |  |  |  |  |  |  |  |  |  |
| *w*^1118^; *Canton-S* | Male | Mechanical | Day | 5 | 3.16 | 0.29 | 0.9171 | 1.72 | N/A |
|  |  | stimulus | Error | 354 | 10.82 | -- | -- |  |  |
|  |  | (Night) |  |  |  |  |  |  |  |
|  |  |  |  |  |  |  |  |  |  |
| *Canton-S* | Female | Mechanical | Day | 5 | 11.06 | 1.80 | 0.1115 | 1.32 | N/A |
|  |  | stimulus | Error | 342 | 6.13 | -- | -- |  |  |
|  |  | (Night) |  |  |  |  |  |  |  |
|  |  |  |  |  |  |  |  |  |  |
| *Canton-S* | Male | Mechanical | Day | 5 | 17.44 | 2.03 | 0.0740 | 1.57 | N/A |
|  |  | stimulus | Error | 336 | 8.59 | -- | -- |  |  |
|  |  | (Night) |  |  |  |  |  |  |  |
|  |  |  |  |  |  |  |  |  |  |
| *Oregon* | Female | Mechanical | Day | 5 | 2.81 | 0.26 | 0.9360 | 1.76 | N/A |
|  |  | stimulus | Error | 342 | 10.94 | -- | -- |  |  |
|  |  | (Night) |  |  |  |  |  |  |  |
|  |  |  |  |  |  |  |  |  |  |
| *Oregon* | Male | Mechanical | Day | 5 | 4.34 | 0.26 | 0.9340 | 2.29 | N/A |
|  |  | stimulus | Error | 306 | 16.62 | -- | -- |  |  |
|  |  | (Night) |  |  |  |  |  |  |  |
|  |  |  |  |  |  |  |  |  |  |
| 22-2 | Female | Mechanical | Day | 5 | 35.93 | 2.82 | 0.0162 | 1.86 | 1-6-2-5-3-4 |
|  |  | stimulus | Error | 366 | 13.07 | -- | -- |  |  |
|  |  | (Night) |  |  |  |  |  |  |  |
| 22-2 | Male | Mechanical | Day | 5 | 35.13 | 2.78 | 0.0178 | 1.94 | 6-5-4-1-3-2 |
|  |  | stimulus | Error | 324 | 12.63 | -- | -- |  |  |
|  |  | (Night) |  |  |  |  |  |  |  |
|  |  |  |  |  |  |  |  |  |  |
| *w*^1118^; *Canton-S* | Female | Mechanical | Day | 5 | 31.80 | 6.73 | <0.0001 | 1.13 | 4-5-3-2-6-1 |
|  |  | stimulus | Error | 354 | 4.72 | -- | -- |  |  |
|  |  | (Day) |  |  |  |  |  |  |  |
|  |  |  |  |  |  |  |  |  |  |
| *w*^1118^; *Canton-S* | Male | Mechanical | Day | 5 | 46.77 | 6.19 | <0.0001 | 1.43 | 6-5-4-2-3-1 |
|  |  | stimulus | Error | 360 | 7.56 | -- | -- |  |  |
|  |  | (Day) |  |  |  |  |  |  |  |
|  |  |  |  |  |  |  |  |  |  |
| *Canton-S* | Female | Mechanical | Day | 5 | 3.75 | 1.01 | 0.4127 | 1.01 | N/A |
|  |  | stimulus | Error | 353 | 3.72 | -- | -- |  |  |
|  |  | (Day) |  |  |  |  |  |  |  |
|  |  |  |  |  |  |  |  |  |  |
| *Canton-S* | Male | Mechanical | Day | 5 | 21.16 | 3.14 | 0.0087 | 1.36 | N/A |
|  |  | stimulus | Error | 354 | 6.74 | -- | -- |  |  |
|  |  | (Day) |  |  |  |  |  |  |  |
|  |  |  |  |  |  |  |  |  |  |
| *Oregon* | Female | Mechanical | Day | 5 | 32.47 | 3.53 | 0.0040 | 1.60 | 5-6-4-3-2-1 |
|  |  | stimulus | Error | 348 | 9.19 | -- | -- |  |  |
|  |  | (Day) |  |  |  |  |  |  |  |
|  |  |  |  |  |  |  |  |  |  |
| *Oregon* | Male | Mechanical | Day | 5 | 32.31 | 2.67 | 0.0021 | 1.82 | 3-4-2-6-5-1 |
|  |  | stimulus | Error | 354 | 12.11 | -- | -- |  |  |
|  |  | (Day) |  |  |  |  |  |  |  |
|  |  |  |  |  |  |  |  |  |  |
| 22-2 | Female | Mechanical | Day | 5 | 51.64 | 6.63 | <0.0001 | 1.51 | 1-2-3-4-6-5 |
|  |  | stimulus | Error | 330 | 7.79 | -- | -- |  |  |
|  |  | (Day) |  |  |  |  |  |  |  |
|  |  |  |  |  |  |  |  |  |  |
| 22-2 | Male | Mechanical | Day | 5 | 29.87 | 3.38 | 0.0054 | 1.56 | 4-3-5-6-2-1 |
|  |  | stimulus | Error | 348 | 8.84 | -- | -- |  |  |
|  |  | (Day) |  |  |  |  |  |  |  |
|  |  |  |  |  |  |  |  |  |  |
| *w*^1118^; *Canton-S* | Female | Light | Day | 5 | 31.49 | 3.50 | 0.0051 | 2.45 | 6-5-4-3-2-1 |
|  |  | stimulus | Error | 144 | 8.98 | -- | -- |  |  |
|  |  |  |  |  |  |  |  |  |  |
|  |  |  |  |  |  |  |  |  |  |
| *w*^1118^; *Canton-S* | Male | Light | Day | 5 | 28.41 | 2.82 | 0.0182 | 2.54 | 2-6-3-5-4-1 |
|  |  | stimulus | Error | 150 | 10.06 | -- | -- |  |  |
|  |  |  |  |  |  |  |  |  |  |
|  |  |  |  |  |  |  |  |  |  |
| *Canton-S* | Female | Light | Day | 5 | 14.06 | 2.04 | 0.0756 | 2.02 | N/A |
|  |  | stimulus | Error | 162 | 6.89 | -- | -- |  |  |
|  |  |  |  |  |  |  |  |  |  |
|  |  |  |  |  |  |  |  |  |  |
| *Canton-S* | Male | Light | Day | 5 | 43.39 | 2.09 | 0.0696 | 3.65 | N/A |
|  |  | stimulus | Error | 150 | 20.75 | -- | -- |  |  |
|  |  |  |  |  |  |  |  |  |  |
| *Oregon* | Female | Light | Day | 5 | 18.87 | 0.96 | 0.4440 | 3.42 | N/A |
|  |  | stimulus | Error | 162 | 19.65 | -- | -- |  |  |
|  |  |  |  |  |  |  |  |  |  |
| *Oregon* | Male | Light | Day | 5 | 37.54 | 1.69 | 0.1397 | 3.77 | N/A |
|  |  | stimulus | Error | 150 | 22.18 | -- | -- |  |  |
|  |  |  |  |  |  |  |  |  |  |
| 22-2 | Female | Light | Day | 5 | 55.08 | 2.39 | 0.0406 | 3.77 | 4-3-5-6-2-1 |
|  |  | stimulus | Error | 156 | 23.08 | -- | -- |  |  |
|  |  |  |  |  |  |  |  |  |  |
| 22-2 | Male | Light | Day | 5 | 27.34 | 1.95 | 0.0892 | 2.94 | N/A |
|  |  | stimulus | Error | 156 | 14.03 | -- | -- |  |  |
|  |  |  |  |  |  |  |  |  |  |
|  |  |  |  |  |  |  |  |  |  |

d.f., degrees of freedom; MS, mean squares; *P*, *P* value. Days are ordered from highest to lowest sleep times. Black, blue, and magenta underlines indicate groups of days with similar sleep times, i.e., a group of days with a black underline indicates that the sleep times for those days did not exceed the minimum significant difference.
